# Nutrigenomics approach elucidates health-promoting effects of high vegetable intake in lean and obese men

**DOI:** 10.1007/s12263-013-0343-9

**Published:** 2013-04-18

**Authors:** W. J. Pasman, M. J. van Erk, W. A. A. Klöpping, L. Pellis, S. Wopereis, S. Bijlsma, H. F. J. Hendriks, A. F. M. Kardinaal

**Affiliations:** TNO, P.O. Box 360, 3700 AJ Zeist, The Netherlands

**Keywords:** Health, Inflammation, Obesity, Vegetables, Adipose tissue

## Abstract

We aimed to explore whether vegetable consumption according to guidelines has beneficial health effects determined with classical biomarkers and nutrigenomics technologies. Fifteen lean (age 36 ± 7 years; BMI 23.4 ± 1.7 kg m^−2^) and 17 obese (age 40 ± 6 years; BMI 30.3 ± 2.4 kg m^−2^) men consumed 50- or 200-g vegetables for 4 weeks in a randomized, crossover trial. Afterward, all subjects underwent 4 weeks of energy restriction (60 % of normal energy intake). Despite the limited weight loss of 1.7 ± 2.4 kg for the lean and 2.1 ± 1.9 kg for the obese due to energy restriction, beneficial health effects were found, including lower total cholesterol, LDL cholesterol and HbA1c concentrations. The high vegetable intake resulted in increased levels of plasma amino acid metabolites, decreased levels of 9-HODE and prostaglandin D3 and decreased levels of ASAT and ALP compared to low vegetable intake. Adipose tissue gene expression changes in response to vegetable intake were identified, and sets of selected genes were submitted to network analysis. The network of inflammation genes illustrated a central role for NFkB in (adipose tissue) modulation of inflammation by increased vegetable intake, in lean as well as obese subjects. In obese subjects, high vegetable intake also resulted in changes related to energy metabolism, adhesion and inflammation. By inclusion of sensitive omics technologies and comparing the changes induced by high vegetable intake with changes induced by energy restriction, it has been shown that part of vegetables’ health benefits are mediated by changes in energy metabolism, inflammatory processes and oxidative stress.

**Electronic supplementary material:**

The online version of this article (doi:10.1007/s12263-013-0343-9) contains supplementary material, which is available to authorized users.

## Introduction

Consumption of vegetables is generally considered to be associated with several positive effects on health. It has been shown that low consumption of fruit and vegetables is related to more cardiovascular disease and cancer (Krebs-Smith and Kantor 2001; Lock et al. 2005; Martinez-González et al. 2011; Mosby et al. 2012). Vegetables are a heterogeneous group of our diet with a low energy density and rich in bio-active compounds, such as vitamins, minerals, dietary fibers and phytochemicals like flavonoids and carotenoids (Tomás-Barberán and Robins 1997; Yao et al. 2004; Martinez-González et al. 2011; Mosby et al. 2012).

The recommended intake of vegetables by the Dutch Health Council is 150–200 g daily (Health Council of the Netherlands 2006). However, it has been reported that only 2 % of the Dutch population meets the recommended daily intake of 150 g of vegetables (Health Council of the Netherlands 2006).

Conflicting results of vegetables have been found in epidemiological studies. Studies with large cohorts of subjects, often report reduced risks for certain diseases when vegetable consumption was increased. The World Cancer Research Fund reported in 2007 that diets containing substantial and varied amounts of vegetable and fruit intake (at least 400 g/day) will prevent 20 % or more of all cancer cases (WCRF 2007). Also, cardiovascular disease risks are lower in subjects consuming more fruit and vegetables. In a meta-analysis of Hu, it was found that consumption of green leafy vegetables had protective effects for coronary heart disease (Hu 2003). Cruciferous vegetables and vitamin C rich citrus fruit and juices were found to protect against stroke (Hu 2003). Also, the development of diabetes mellitus may be reduced with vegetable consumption (Bazzano et al. 2008; Villegas et al. 2008). Several specific compounds present in vegetables have been studied for their potential beneficial effects: antioxidants (especially polyphenols, lycopene and vitamin C) as free radical trapper; dietary fiber for its effect on gastro-intestinal functioning and related satiety; and glucosinolates present in the *Brassicaceae* vegetables family (cabbage; Brussels sprouts; cauliflower; broccoli), for their chemopreventive effect against cancer (Hayes et al. 2008). Based on epidemiological data, most national guidelines prescribe consumption of at least 200 g of vegetables and two pieces of fruit daily. The European Prospective Investigation into Cancer and Nutrition (the EPIC study) reported, however, no protective effect of total or specific vegetables intake against different types of cancer, like gastric cancer or urothelial cell carcinoma (Gonzalez et al. 2012; Ros et al. 2012).

Therefore, the inverse relationship of vegetable intake and disease risk is complex, also in relation to obesity. Vegetable consumption correlates with a lower risk of obesity (Martin et al. 2011; Myint et al. 2011), while increased body weight is related to increased risk of cancer (Wolin et al. 2010; La Vecchia et al. 2011), coronary (the Emerging Risk Factors Collaboration, 2011; Yusuf et al. 2005) and metabolic diseases (Boden 2008; Abdullah et al. 2010). Weight loss of 5–10 % of the initial body weight has been shown to improve risk profiles of diseases related to obesity (Wing et al. 1992; Tuomilehto et al. 2001). Also, weight loss due to caloric restriction will lead to changes in adipose tissue (Márquez-Quiñones et al. 2010; Bouchard et al. 2010).

Measuring the effects of a nutritional intervention such as increased vegetable intake is difficult, as the effects are likely to be subtle and variable across subjects. We and others have demonstrated that integration of omics technologies in nutrition research can yield more insights into effects of nutrition on health-related physiological processes (van Erk et al. 2008; Radonjic et al. 2009; Bakker et al. 2010).

The mechanisms of action for the vegetable intervention and the energy restriction on health may differ. High vegetable intake has been suggested to be mainly anti-inflammatory due to its high antioxidant content (Calder et al. 2011). It is also possible that certain phenolic compounds of fruits and vegetables may also have some caloric restriction mimetic effects if they activate sirtuin deacetylases (Chen et al. 2008). Energy restriction will limit the effects of energy-driven processes like oxidative phosphorylation, mitochondrion activity, generation of precursor metabolites and energy (Calder et al. 2011).

In the present study, the effect of high vegetable consumption was investigated in a crossover trial by comparing this to low vegetable consumption in both lean and obese subjects. An energy-restricted dietary intervention was conducted in a parallel design after the crossover part, to examine whether part of the effects of vegetables may be attributed to similar mechanisms.

We performed a large-scale assessment of changes in a range of blood parameters using sensitive (omics) techniques. Analyses included the profiling of cytokines, amino acids and oxylipins to monitor the effect of the vegetable intervention on processes related to inflammation, oxidative stress, beta-oxidation and protein and amino acid metabolism. Furthermore, we assessed effects on adipose tissue by transcriptome analysis of adipose tissue biopsies. Interpretation of effects of high vegetable intake in light of effects induced by energy restriction gives additional insight into health-promoting effects of vegetables.

## Methods

### Study design

The study was a combination of a crossover trial for the vegetable intervention and a parallel setup for the third period. This was an energy-restricted diet intervention for all subjects.

Each intervention period lasted 4 weeks. With the high vegetable treatment, subjects consumed 200 g of vegetables daily, and with the low vegetable treatment, subjects consumed 50 g of vegetables daily. Subjects were free to consume their vegetables with lunch and/or with dinner. The vegetables were provided weekly by TNO; about ten different, most popular vegetables could be chosen; fresh vegetables for the first and second day of the week (tomatoes; beet; onions; cauliflower) and canned vegetables for the rest of the week [peas; carrots; French beans; broad beans; corn (mixture)]. Each type of vegetable could be chosen only once a week to guarantee a variation in the diet. Intake of fruit was limited to one piece a day to prevent confounding effects. Further food intake and lifestyle were maintained as usual.

The amount of food consumed during the energy-restricted diet intervention was based on the food intake diaries during the crossover interventions. All subjects were instructed to consume about 60 % of their normal energy intake. A dietitian advised the subjects and monitored compliance.

The energy-restricted intervention was added as a positive control for vegetable treatment because of its known beneficial effects on health. Due to the expected weight loss in the energy-restricted period, no complete crossover design was applied in the study, because the effect of weight loss is expected to effect the next treatment.

The study was conducted at TNO (Zeist, the Netherlands). The study was approved by an independent centralized ethics committee (METOPP, Tilburg, the Netherlands) and is registered at Clinicaltrials.gov, number NCT 00959790.

After inclusion, subjects were randomly allocated to entry number and study treatment (vegetable dose order), in such a way that age and BMI were equally divided among treatment groups.

The study was designed as an open study, so subjects and investigators were aware of the dosage of vegetables consumed. The energy intake was different between the two vegetable treatments: in the 200-g vegetable intervention, about 30–70 kcal was consumed with vegetable consumption, whereas this was 15–35 kcal for vegetables in the 50-g vegetable intervention. This difference in energy intake was part of the intervention (the difference in energy intake is about 15–55 kcal/day; 0.5–1.0 % of the habitual daily energy intake (about 3,000 kcal for males/day)). No energy compensation occured as could be concluded from the maintenance of body weights.

The difference of 150-g vegetable consumption affected the intake of vitamins and minerals; vitamin C (26 mg), vitamin B (total about 0.4 mg), lycopene (153 μg), retinol equivalents (360 RE), lutein (1,473 μg), sodium (71 mg), potassium (345 mg), calcium (65 mg), phosphorus (69 mg), magnesium (23 mg) and iron (69 mg) differed mostly between the two treatment dosages. The subjects were instructed to maintain their habitual body weight, food pattern and physical activity pattern in the first two intervention periods and to consume only the vegetables provided by TNO. To monitor food intake and compliance, subjects wrote down their food intake for 3 days in the second and fourth week of each treatment. Daily compliance of vegetable consumption was registered as well. On the day before each test day, all subjects consumed French beans as a standardized meal to reduce variation in the study samples.

### Subjects

We selectively recruited apparently healthy, lean and overweight/obese, non-smoking male subjects. Sixty-three subjects came to TNO for an oral information session. Forty-eight subjects signed an informed consent. Thirty-nine subjects were eligible, and 34 subjects were included. Two subjects dropped out during the study for non-study-related reasons; therefore, the study was completed with 32 subjects. Fifteen subjects were lean (BMI 23.4 ± 1.7 kg m^−2^), and seventeen subjects were overweight/obese (BMI 30.3 ± 2.4 kg m^−2^). Subjects were aged between 18 and 45 years, exercised less than 2.5 h a week and were used and liked to eat vegetables. Subjects received financial compensation for their participation.

### Experimental protocol

At the end of each 4-week intervention period, a test day was conducted (day 29, 57 and 85).

On each test day, a fasted blood sample was obtained. Physical variables like body weight, waist and hip circumferences and blood pressure were measured.

A standard breakfast was provided after blood sampling. A maximal exercise test was performed after breakfast consumption. The results of the exercise tests will be presented elsewhere.

### Blood and urine clinical chemistry analyses

Blood samples were obtained from the antecubital vein of the forearm and collected in tubes containing clot activator for serum and in tubes containing EDTA for plasma (Vacutainer Systems, Becton–Dickinson, Plymouth, U.K.). Blood was centrifuged for 15 min at 2,000*g* and 4 °C within 30 min after collection. Plasma and serum samples were stored at <−70 °C until analysis.

Serum clinical chemistry tests included the measurement of glucose, triacylglycerol (TG), free fatty acids (FFA), glycerol, insulin, HbA1c, total cholesterol and HDL-cholesterol, γ-GT, ALAT, ASAT, ALP, hamatology, total bilirubin, urea, uric acid, creatinine, lactate, creatine kinase and myoglobin and were performed using Olympus analytical equipment and reagents (Olympus AU400 clinical chemistry analyzer; Olympus-Diagnostica Europe, Hamburg, Germany).

Plasma samples of 10 lean and 10 obese subjects were used for multiplex analysis of 6 inflammatory proteins: GM-CSF, IFNgamma, IL-1beta, IL-6, IL-8 and TNF-alpha (Mesoscale Discovery). Plasma levels of IFNgamma and IL-1beta were too low and could not be detected.

### Plasma profiling of oxylipins and amino acids

To monitor the effect of the vegetable intervention on processes related to inflammation, oxidative stress, beta-oxidation and protein and amino acid metabolism, blood samples were collected for liquid chromatography–mass spectrometry (LC–MS) analysis of oxylipins and amino acids after an overnight fast at the end of the 4-week low and high vegetable interventions. For oxylipin analysis, blood was collected in tubes containing EDTA. These blood samples were directly supplemented with an inhibitor cocktail containing paraoxon, butylated hydroxytoluene (BHT), indomethacin and phenyl methyl sulfonylfluoride (PMSF) with the following proportions 1 BHT: 1 Indomethacin: 10 Paraoxon: 1 PMSF to prevent oxylipin oxidation and breakdown. For amino acid analysis, blood was collected in tubes containing Li-heparin as anti-coagulant. Blood was put on ice till centrifugation. All samples were centrifuged within 30 min after collection. Blood was centrifuged for 15 min at 2,000*g* and 4 °C. Plasma and serum samples were stored at <−70 °C until analysis.

The LC–MS/MS method used for the measurement of plasma oxylipins and the LC–MS method used for the measurement of a broad range of plasma amino acids and derivatives were identical to the methods reported by, respectively, Balvers et al. (2012) and Rosenling et al. (2009). The samples were analyzed in randomized order. For amino acids, data for each subject were corrected for the recovery of the internal standard for injection. The performance of the applied metabolic profiling platforms was assessed through the frequent analysis of the quality control sample. Finally, the oxylipin data set contained 21 metabolites and the amino acid data set consisted of 51 metabolites (see Supplementary File 1).

### Fat biopsy

After 3 weeks of treatment intervention, at day 22, 50 and 78, an abdominal, subcutaneous fat sample was obtained after an overnight fast from all subjects. Human abdominal fat biopsies were taken according to Kolaczynski et al. (1994). Briefly, subcutaneous adipose tissue was obtained by needle aspiration under local anesthesia using two syringes of 50 mL 8.4 % NaHCO3, 0.05 % lidocaine and 1.0 mg/ml epinephrine. The two syringes used for fat collection contained in total about 10 mL of adipose tissue. The adipose tissue was divided over cryovials, immediately frozen in liquid nitrogen and stored at −80 °C for RNA isolation (van Erk et al. 2008).

### Transcriptomics

Total RNA was extracted from adipose tissue samples from 10 lean and 10 obese subjects (a subset of subjects in both groups) using RNAzol (Campro Scientific, Veenendaal, the Netherlands) and glass beads according to the manufacturer’s instructions. RNA was subjected to a cleanup step using NucleoSpin columns (Macherey–Nagel, Germany).

Quality control, RNA labeling, hybridization and data extraction were performed at ServiceXS (Leiden, the Netherlands). RNA concentration was measured using a Nanodrop ND-1000 spectrophotometer (Nanodrop Technologies, Wilmington, DE, USA). The RNA quality and integrity was determined using Lab-on-Chip analysis on an Agilent 2100 Bioanalyzer (Agilent Technologies, Inc., Santa Clara, CA, USA). The Ambion Illumina total prep 96 kit was used to create biotinylated cRNA from the RNA samples. For the hybridization, wash and stain steps and scanning of the Illumina BeadChips the Illumina protocol: “Whole-Genome Gene Expression Direct Hybridization Assay” was followed strictly. The amount of biotinylated cRNA hybridized onto the HumanHT-12 v.3 Expression BeadChip was 750 ng. In total, 60 RNA samples from adipose tissue were of sufficient quality and subjected to array analysis.

Due to scanning problems for one sample, data of 19 subjects were included in further analysis.

Data were extracted using GenomeStudio. Quality control and normalization (quantile) of microarray data was performed in Genespring GX11.0. Non-expressed genes were removed by filtering on detection value (*p* value ≥0.99 in at least one sample). This resulted in gene expression values for 21,525 genes with unique identifiers.

The microarray data will be made available in the GEO database (http://www.ncbi.nlm.nih.gov/geo/).

### Statistics

The number of subjects participating in the present study was based on the main health biomarker “oxidative stress.” This parameter was tested with a maximal exercise performance on a cycle ergometer, and therefore, the number of subjects reported in the review of Fisher-Wellman and Bloomer (2009) was used for the determination of group size in the present study.

The vegetable interventions (health effect) were tested according to the crossover design and the energy-restricted period according to the parallel design. For the crossover design, all variables were compared between treatments and weight class with a mixed model that included treatment, weight class and the interaction between weight class and treatment as fixed factors and period and subject as random factors. If the analysis indicated an interaction between weight class (lean and obese) and treatment (*p* < 0.05), comparisons between means of the parameters were performed using a 2-sided Student *t* test. A mixed model on the difference between last measurement in the second intervention period (last measurement in crossover design) and the measurement after the third intervention (energy restriction) was used to investigate the effect of the caloric restriction. Although the statistics is performed on the changes resulting from energy restriction (comparison of values after energy restriction period to values before energy restriction period), to control for prior vegetable dose (high or low), in the tables, the absolute values are presented.

If necessary, data were log-transformed and statistical outliers (defined as an observation for which the absolute residual was three times higher than the square root of the model error) were removed. In all statistical tests performed, the null hypothesis (no effect) was rejected at the 0.05 level of probability.

Expression data of the transcriptomics were log-transformed (base 2). To reveal the effect of high vegetables, a two-way ANOVA was applied with the following factors: group (lean/obese), treatment (high vegetables/low vegetables) and interaction. If the ANOVA assumptions were not met, data were rank-transformed and the ANOVA model was applied on rank-transformed data. In all statistical tests performed, the null hypothesis (no effect) was rejected at the 0.05 level of probability. In addition to a threshold on *p* value, a threshold was applied to fold change induced by intervention. Genes were used for biological interpretation if the *p* value threshold was passed and if the intervention resulted in more than 25 % expression difference in same direction in at least 60 % of the subjects (all up or all down).

Pearson correlation coefficients were calculated between gene expression changes (2log ratio for after vs. before energy restriction) and body weight change. Genes with correlation coefficient above 0.7 or below −0.7 were selected and submitted to enrichment analysis in DAVID (Huang et al. 2009). When correlation was contributable to one outlier value, these were not taken into account for interpretation. Statistical analyses were performed using the SAS statistical software package (SAS version 8.2, SAS Institute, Cary, NC, USA).

### Biological interpretation

Gene enrichment analysis was performed using DAVID Functional enrichment tools (http://david.abcc.ncifcrf.gov/) (Huang et al. 2009), selecting enriched gene groups (*p* < 0.05) with 5 or more differentially expressed genes. Network analysis of genes, proteins and metabolites was performed in MetaCore version 6.7 (GeneGo Inc., St. Joseph, MI, USA), using the shortest path algorithm (max 2 steps). Prostaglandin D3 and Methionine sulfoxide were not present in the MetaCore database and were therefore excluded from network analysis.

## Results

This study aimed to investigate the effect of high vegetable consumption in lean and obese subjects compared to low vegetable consumption. The baseline characteristics and fasting blood data of the subjects are shown in Table 1. Not only body weight, BMI, waist circumference and waist–hip ratio were different between lean and obese group, but also levels of insulin, total cholesterol and LDL cholesterol. All these parameters were significantly higher in the obese compared to the lean group.

**Table 1:** Baseline characteristics and fasting blood data of the lean (*n* = 15) and obese (*n* = 17) men who completed the study (mean ± SD is presented)

Parameter	Lean (*n* = 15)	Obese (*n* = 17)	*p* value
Age (years)	36 ± 7	40 ± 6	N.S.
Body weight (kg)	82.9 ± 9.2	101.4 ± 11.4	<0.001
BMI (kg m^−2^)	23.4 ± 1.7	30.3 ± 2.4	<0.001
Waist circumference (cm)	88.9 ± 4.8	105.6 ± 6.6	<0.001
Waist–hip ratio	0.89 ± 0.04	0.97 ± 0.04	<0.001
Systolic blood pressure (mm Hg)	120 ± 13	127 ± 12	N.S.
Diastolic blood pressure (mm Hg)	79 ± 9	83 ± 11	N.S.
Glucose (mmol/L)	5.5 ± 0.4	5.6 ± 0.4	N.S.
Insulin (mmol/L)	6.2 ± 2.4	12.9 ± 6.5	<0.05
HbA1c (%)	5.1 ± 0.2	5.0 ± 0.2	N.S.
Total cholesterol (mmol/L)	4.9 ± 0.6	5.7 ± 0.7	<0.05
HDL-cholesterol (mmol/L)	1.3 ± 0.3	1.2 ± 0.2	+
LDL-cholesterol (mmol/L)	3.0 ± 0.4	3.6 ± 0.5	<0.05

+ Represents a tendency (*p* < 0.1); *N.S.* means not significant

### Compliance in the study

Compliance of vegetable consumption was evaluated at the end of the vegetable treatments, by registration of the daily intake of the vegetables. If study substances were not consumed or the amount was not equal to the required 50 or 200 g, this was registered as a protocol deviation. In the two times 4-week treatment period, the 32 subjects were supposed to consume 1,792 portions of vegetables in total. Twenty vegetable consumption protocol deviations (more or less vegetable intake, evenly divided (some forgot, some consumed more)) were registered and therefore compliance for vegetable intake was 98.9 %. When the 16 fruit intake deviations were taken into account as well (mainly consumption of two pieces of fruit in stead of one) (in total 36 fruit and vegetable consumption deviations were reported), compliance was still 98.0 %. Compliance to vegetable treatment was therefore qualified as good.

During the energy-restricted intervention, subjects were instructed to consume about 30–40 % less energy daily (individual advices were given by the dietitian), which was calculated to result in ~1 kg weight loss per week. The caloric restriction intervention resulted in weight loss in lean and obese subjects (−1.7 ± 2.4 kg in lean and −2.1 ± 1.9 kg in obese). The weight loss was significant over time (*p* < 0.0001), but not different between the lean and obese. This amount of weight loss was about 50 % of the amount expected after 4 weeks of energy restriction; therefore, the compliance was also about 50 %. The compliance during the energy-restricted diet period was therefore qualified as moderate.

### Plasma markers

The caloric restriction intervention was used as a positive control condition to evaluate the effects found with the vegetable interventions. Tables 2 and 3 indicate the plasma marker changes in response to the caloric restriction and the vegetable intervention, respectively. Overall, the changes were not different between lean and obese subjects; very few markers showed a significant different response in obese compared to lean subjects (significant interaction in ANOVA).

**Table 2:** Changes in blood parameters after the energy-restricted (ER) intervention (mean ± SD is presented)

Treatment	Lean (*n* = 15)		Obese (*n* = 17)		*p* value^#^
	Before ER	After ER	Before ER	After ER	
Glucose (mmol/L)	5.4 ± 0.3	5.5 ± 0.3	5.7 ± 0.3	5.7 ± 0.4	N.S.
Insulin (mU/L)	5.9 ± 3.3	5.0 ± 2.7	12.7 ± 6.1	11.0 ± 6.8	N.S.
HbA1c (%)	5.1 ± 0.2	5.0 ± 0.2	5.1 ± 0.2	5.0 ± 0.1	<0.05
Total cholesterol (mmol/L)	5.2 ± 0.7	4.7 ± 0.7	6.0 ± 0.7	5.4 ± 0.5	<0.05
HDL-cholesterol (mmol/L)	1.3 ± 0.4	1.3 ± 0.3	1.2 ± 0.2	1.1 ± 0.2	N.S.
LDL-cholesterol (mmol/L)	3.3 ± 0.6	2.9 ± 0.6	3.9 ± 0.6	3.5 ± 0.4	<0.05
Ratio cholesterol/HDL	4.3 ± 1.2	3.8 ± 0.9	5.3 ± 0.9	4.9 ± 0.9	<0.05
Triacylglycerol (mmol/L)	1.4 ± 0.7	1.1 ± 0.5	2.2 ± 1.2	2.1 ± 1.4	N.S.
γ-GT (U/L)	23.4 ± 11.2	18.2 ± 8.0	33.9 ± 12.8	27.5 ± 12.5	<0.05
ALAT (U/L)	12 ± 4	12 ± 4	19 ± 11	19 ± 11	N.S.
ASAT (U/L)	21 ± 4	20 ± 4	26 ± 8	24 ± 6	+
ALP (U/L)	64 ± 12	60 ± 10	67 ± 18	65 ± 14	N.S.
TNF-α (pg/mL)^a^	0.97 ± 0.29	0.88 ± 0.18	1.08 ± 0.34	1.11 ± 0.39	N.S.

^#^ *p* values for treatment effect in 2-way ANOVA, interaction effects (BMI-category × treatment) were not significant

^+^ *p* < 0.1 (tendency)

^a^Two lean subjects were excluded from the TNF-α dataset because they were outliers, *ER* energy restriction

**Table 3:** Changes in blood clinical chemistry parameters after the vegetable intervention (mean ± SD is presented)

Treatment	Lean (*n* = 15)		Obese (*n* = 17)		*p* value^#^
	50-g Veg	200-g Veg	50-g Veg	200-g Veg	
Glucose (mmol/L)	5.4 ± 0.3	5.4 ± 0.3	5.7 ± 0.4	5.9 ± 0.1	N.S.
Insulin (mU/L)	5.5 ± 2.6	5.9 ± 3.2	11.0 ± 5.2	13.4 ± 6.7	N.S.
HbA1c (%)	5.1 ± 0.2	5.1 ± 0.2	5.1 ± 0.1	5.1 ± 0.2	N.S.
Total cholesterol (mmol/L)	5.1 ± 0.7	5.2 ± 0.6	6.0 ± 0.8	5.8 ± 0.7	N.S.
HDL-cholesterol (mmol/L)	1.3 ± 0.3	1.3 ± 0.3	1.2 ± 0.2	1.1 ± 0.2	N.S.
LDL-cholesterol (mmol/L)	3.2 ± 0.6	3.4 ± 0.4	3.9 ± 0.6	3.8 ± 0.6	N.S.
Ratio cholesterol/HDL	4.2 ± 1.2	4.2 ± 0.9	5.3 ± 0.9	5.3 ± 0.9	N.S.
Triacylglycerol (mmol/L)	1.3 ± 0.7	1.2 ± 0.5	2.2 ± 1.2	2.2 ± 1.7	N.S.
γ-GT (U/L)	23.3 ± 10.9	22.4 ± 10.8	34.3 ± 13.1	32.9 ± 11.6	N.S.
ALAT (U/L)	13 ± 4	11 ± 4	21 ± 15	21 ± 10	N.S.
ASAT (U/L)	21 ± 4	20 ± 4	26 ± 8	25 ± 8	<0.05
ALP (U/L)	63 ± 11	61 ± 11	67 ± 18	64 ± 15	<0.05
TNF-α (pg/mL)^a^	1.03 ± 0.28	0.84 ± 0.17	1.11 ± 0.41	1.09 ± 0.36	0.0067*

^#^ *p* values for treatment effect in 2-way ANOVA, interaction effects (BMI-category × treatment) were not significant

* *p* value for vegetable effect in lean subjects (post hoc test); *p* value for interaction (BMI-category × treatment) was 0.0491

^a^Two lean subjects were excluded from the TNF-α dataset because they were outliers; g, gram and Veg, vegetable

Blood levels of total cholesterol, LDL cholesterol, ratio of cholesterol/HDL, γ-GT, ASAT and HbA1c decreased significantly in response to the energy restriction diet in both lean and obese groups (Table 2).

Only fasting ASAT and ALP levels were lower after consumption of 200-g vegetables compared to 50-g vegetables in both lean and obese subjects (Table 3). TNF-α, an inflammation marker, showed an interaction between weight class and treatment: TNF-α levels were significantly lower in lean men after 200 g of vegetable treatment compared to the 50 g of vegetable treatment.

The inventory of health-promoting effects of vegetables in lean and obese subjects included measurements of plasma metabolites related to amino acids and derivatives (*n* = 51) and oxylipins (*n* = 21) (see Supplementary File 1) and measurement of 4 inflammatory plasma proteins (IL-6, IL-8, TNF-α and GM-CSF). The plasma metabolites with a significant change related to vegetable intake are summarized in Table 4. Intake of 200 g compared to 50-g vegetables resulted in increased plasma levels of asparagine (+6.8 %), leucine (+5.0 %), methionine sulfoxide (+15 %) and threonine (+5.9 %). Levels of 9-HODE (−21.4 %) and prostaglandin D3 (−16.7 %) were significantly lower in response to the 200-g vegetable intake compared to 50-g vegetable intake. Uniquely in obese subjects, levels of 15-HETE were lowered (−31.7 %). Vegetable intake did not result in significant changes in inflammatory plasma proteins IL-6, IL-8, TNF-α and GM-CSF in this selected group of subjects and analysis.

**Table 4:** Significant changes in blood metabolites after the vegetables intervention (mean ± SD is presented)

Treatment	Lean (*n* = 15)		Obese (*n* = 17)		*p* value^#^
	50-g Veg	200-g Veg	50-g Veg	200-g Veg	
Asparagine (Asn)	0.30 ± 0.06	0.32 ± 0.06	0.28 ± 0.05	0.30 ± 0.07	0.0418
Leucine (Leu)	1.61 ± 0.13	1.65 ± 0.15	1.83 ± 0.33	1.96 ± 0.36	0.0224
Methionine sulfoxide 1	1.9 × 10^−3^ ± 8.8 × 10^−4^	2.2 × 10^−3^ ± 2.9 × 10^−4^	1.9 × 10^−3^ ± 3.7 × 10^−4^	2.2 × 10^−3^ ± 5.7 × 10^−4^	0.0308
Methionine sulfoxide 2	2.7 × 10^−3^ ± 1.2 × 10^−3^	2.9 × 10^−3^ ± 4.2 × 10^−4^	2.6 × 10^−3^ ± 6.7 × 10^−4^	3.1 × 10^−3^ ± 7.2 × 10^−4^	0.0455
Threonine (Thr)	2.32 ± 0.25	2.37 ± 0.28	2.26 ± 0.39	2.49 ± 0.53	0.0205
9-HODE	2.62 ± 0.83	2.20 ± 1.30	3.00 ± 1.31	2.21 ± 0.46	0.0187
PGD-3	0.024 ± 0.024	0.020 ± 0.021	0.017 ± 0.016	0.013 ± 0.016	0.0408
15-HETE	0.08 ± 0.03	0.10 ± 0.05	0.12 ± 0.06	0.08 ± 0.04	0.0263*

*G* gram; *Veg* vegetable

^*#*^ *p* values for treatment effect in 2-way ANOVA, interaction effects (BMI-category × treatment) were not significant

* *p* value for vegetable effect in obese subjects (post hoc test); *p* value for interaction (BMI-category × treatment) was 0.006

### Effects of increased vegetable intake on adipose tissue

We aimed to investigate the effect of increased vegetable intake on adipose tissue in lean and obese subjects by gene expression analysis. Overall, limited gene expression changes were observed due to high vegetable intake compared to low vegetable intake: 323 genes were affected in obese subjects and 532 genes in lean. The differentially expressed genes in lean subjects were enriched for genes involved in immune and inflammatory response. In lean subjects, high vegetable consumption resulted in reduced expression of adipose tissue genes IL-8 and NFKB2 and increased expression of adipose tissue genes complement component 3 (C3) and NFKB inhibitor NFKBIB. The Table in Supplementary File 2 lists the 40 differentially expressed genes in lean subjects annotated to these processes. In obese subjects, no gene ontology biological processes were enriched in the set of 323 genes.

**Fig. 1:**
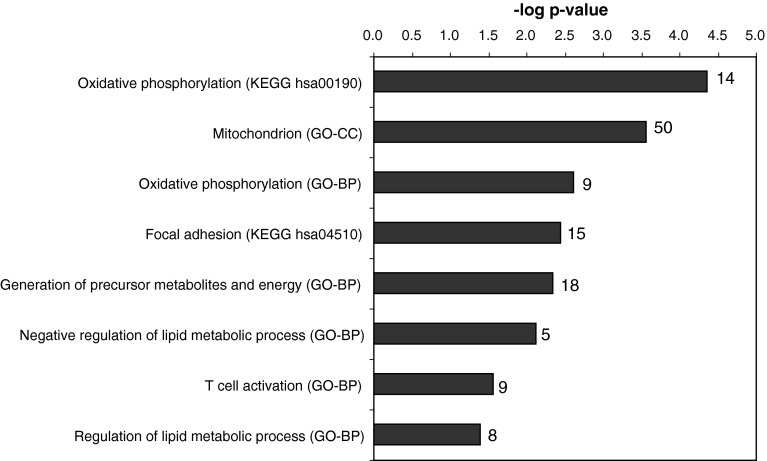
Enrichment analysis of 641 genes with high correlation to body weight change in 10 obese subjects

We investigated to what extent adipose tissue gene expression changes in response to caloric restriction were associated with weight loss. Weight loss in response to caloric restriction was similar to lean and obese subjects. However, very limited gene expression changes were associated with amount of weight loss when all subjects were taken together (6 genes with *r* > |0.7|, data not shown). Next, lean and obese subjects were analyzed separately, to check for correlations within these groups. In the group of obese subjects, 641 genes showed high correlation (*r* > |0.7|) to body weight change compared to 303 genes in the group of lean subjects. Enrichment analysis showed that in obese subjects, body weight change due to caloric restriction intervention correlated with gene expression responses in processes of energy metabolism (oxidative phosphorylation, mitochondrion, generation of precursor metabolites and energy, regulation of lipid metabolism), focal adhesion and inflammation (T cell activation) in adipose tissue (Fig. 1). In lean subjects, mainly gene expression changes related to inflammatory response processes correlated with body weight change. However, these were contributable to outlier values for 1 subject and therefore not taken into account further.

Although we were not able to detect enriched processes related to vegetable intake in obese subjects based on the set of 323 differentially expressed genes (see above), we now investigated whether in obese subjects, the increased vegetable consumption did affect expression of genes annotated to the processes that were associated with weight loss in obese subjects. Table 5 lists differentially expressed genes (consistent expression change, i.e. >25 % up or >25 % down in at least 6 subjects, combined with *p* < 0.05 in ANOVA) in response to high vegetable intake compared to low vegetable intake involved in energy metabolism, adhesion and inflammation in obese subjects. This resulted in 16 genes for energy metabolism, 18 genes for cell adhesion and 19 genes for inflammation.

**Table 5:** Expression changes in obese subjects in response to high vegetable intake compared to low vegetable intake related to inflammation, energy metabolism and adhesion

Definition	Symbol	Probe_Id	Entrez Gene ID	Mean 2log ratio	
Energy metabolism					
Glutamate-ammonia ligase (glutamine synthetase) (GLUL), transcript variant 3, mRNA	GLUL	ILMN_1765208	2752	0.67	a
Runt-related transcription factor 1; translocated to, 1 (cyclin D-related) (RUNX1T1), transcript variant 3, mRNA	RUNX1T1	ILMN_1710522	862	0.65	a
Peroxisome proliferator-activated receptor gamma, coactivator-related 1 (PPRC1), mRNA	PPRC1	ILMN_1796210	23082	0.65	b
SLIT-ROBO Rho GTPase activating protein 2 (SRGAP2), transcript variant 1, mRNA	SRGAP2	ILMN_1759549	23380	0.57	b
Pyruvate carboxylase (PC), nuclear gene encoding mitochondrial protein, transcript variant 3, mRNA	PC	ILMN_1736689	5091	0.44	a
FERM domain containing 6 (FRMD6), transcript variant 2, mRNA	FRMD6	ILMN_2330787	122786	0.42	b
Solute carrier family 25, member 29 (SLC25A29), nuclear gene encoding mitochondrial protein, mRNA	SLC25A29	ILMN_2350801	123096	0.40	b
Solute carrier family 25 (mitochondrial carrier, brain), member 14 (SLC25A14), nuclear gene encoding mitochondrial protein, transcript variant long, mRNA	SLC25A14	ILMN_1743770	9016	0.39	a
Acyl-Coenzyme A dehydrogenase, C-2 to C-3 short chain (ACADS), nuclear gene encoding mitochondrial protein, mRNA	ACADS	ILMN_1795104	35	−0.27	b
DNA replication helicase 2 homolog (yeast) (DNA2), mRNA	DNA2	ILMN_2282959	1763	−0.57	b
Protein kinase C, alpha binding protein (PRKCABP), mRNA	PRKCABP	ILMN_1708159	9463	−0.67	a
Apolipoprotein O-like (APOOL), mRNA	APOOL	ILMN_1777483	139322	−0.68	a
Acyl-CoA synthetase medium-chain family member 5 (ACSM5), mRNA	ACSM5	ILMN_1801698	54988	−0.71	a
Estrogen receptor 2 (ER beta) (ESR2), transcript variant b, mRNA	ESR2	ILMN_2390457	2100	−0.74	a
PREDICTED: coiled-coil domain containing 56 (CCDC56), misc RNA	CCDC56	ILMN_1789786	28958	−0.86	a
Component of oligomeric golgi complex 8 (COG8), mRNA	COG8	ILMN_1725246	84342	−1.09	b
Adhesion					
Presenilin 1 (Alzheimer disease 3) (PSEN1), mRNA	PSEN1	ILMN_1796669	5663	1.13	a
PREDICTED: p21 (CDKN1A)-activated kinase 2 (PAK2), mRNA	PAK2	ILMN_1659878	5062	0.96	a
Reelin (RELN), transcript variant 1, mRNA	RELN	ILMN_1753005	5649	0.89	b
LIM and senescent cell antigen-like domains 1 (LIMS1), mRNA	LIMS1	ILMN_2381037	3987	0.71	b
Dystonin (DST), transcript variant 1e, mRNA	DST	ILMN_1675992	667	0.70	a
Rap guanine nucleotide exchange factor (GEF) 1 (RAPGEF1), transcript variant 2, mRNA	RAPGEF1	ILMN_1678799	2889	0.50	a
Protocadherin 9 (PCDH9), transcript variant 2, mRNA	PCDH9	ILMN_2379626	5101	0.46	b
Phosphoglucomutase 5 (PGM5), mRNA	PGM5	ILMN_2271149	5239	0.45	b
Periostin, osteoblast specific factor (POSTN), mRNA	POSTN	ILMN_1790761	10631	0.44	b
Claudin 15 (CLDN15), transcript variant 2, mRNA	CLDN15	ILMN_1708267	24146	0.38	a
PREDICTED: p21 (CDKN1A)-activated kinase 2 (PAK2), mRNA	PAK2	ILMN_1676385	5062	0.38	a
Polycystic kidney and hepatic disease 1 (autosomal recessive) (PKHD1), transcript variant 1, mRNA	PKHD1	ILMN_1720034	5314	−0.30	b
Cadherin, EGF LAG seven-pass G-type receptor 1 (flamingo homolog, Drosophila) (CELSR1), mRNA	CELSR1	ILMN_1694482	9620	−0.50	b
v-akt murine thymoma viral oncogene homolog 3 (protein kinase B, gamma) (AKT3), transcript variant 2, mRNA	AKT3	ILMN_1733598	10000	−0.59	b
Junction plakoglobin (JUP), transcript variant 2, mRNA	JUP	ILMN_2366864	3728	−0.67	a
Myeloid/lymphoid or mixed-lineage leukemia (trithorax homolog, Drosophila); translocated to, 4 (MLLT4), transcript variant 3, mRNA	MLLT4	ILMN_1746277	4301	−0.77	b
Cadherin-like 24 (CDH24), transcript variant 1, mRNA	CDH24	ILMN_1693928	64403	−0.83	a
Rap guanine nucleotide exchange factor (GEF) 1 (RAPGEF1), transcript variant 1, mRNA	RAPGEF1	ILMN_1695282	2889	−0.93	b
Inflammation					
Presenilin 1 (Alzheimer disease 3) (PSEN1), mRNA	PSEN1	ILMN_1796669	5663	1.13	a
Cathepsin C (CTSC), transcript variant 2, mRNA	CTSC	ILMN_1689086	1075	0.78	b
Membrane-associated ring finger (C3HC4) 8 (MARCH8), transcript variant 6, mRNA	MARCH8	ILMN_2341626	220972	0.78	a
Interleukin 7 receptor (IL7R), mRNA	IL7R	ILMN_2342579	3575	0.73	a
Interleukin 8 (IL8), mRNA	IL8	ILMN_2184373	3576	0.66	a
Tumor necrosis factor receptor superfamily, member 13C (TNFRSF13C), mRNA	TNFRSF13C	ILMN_1731742	115650	0.63	a
Colony stimulating factor 1 (macrophage) (CSF1), transcript variant 4, mRNA	CSF1	ILMN_1805930	1435	0.52	b
Epstein-Barr virus-induced 3 (EBI3), mRNA	EBI3	ILMN_1802653	10148	0.51	a
Apolipoprotein B mRNA editing enzyme, catalytic polypeptide-like 3F (APOBEC3F), transcript variant 1, mRNA	APOBEC3F	ILMN_1710726	200316	0.49	b
Neutrophil cytosolic factor 1 (NCF1), mRNA	NCF1	ILMN_1697309	653361	0.38	a
Cathepsin C (CTSC), transcript variant 1, mRNA	CTSC	ILMN_2242463	1075	0.34	b
B and T lymphocyte associated (BTLA), transcript variant 1, mRNA	BTLA	ILMN_1778536	151888	−0.19	a
Chemokine (C-X-C motif) receptor 5 (CXCR5), transcript variant 2, mRNA	CXCR5	ILMN_2337931	643	−0.42	a
Interleukin 1 receptor, type II (IL1R2), transcript variant 2, mRNA	IL1R2	ILMN_1772131	7850	−0.60	b
Lymphocyte transmembrane adaptor 1 (LAX1), mRNA	LAX1	ILMN_1769782	54900	−0.61	a
Serum amyloid A1 (SAA1), transcript variant 1, mRNA	SAA1	ILMN_1701017	6288	−0.68	b
Virus-induced signaling adapter (VISA), mRNA	VISA	ILMN_2131493	57506	−0.69	a
Tumor necrosis factor (ligand) superfamily, member 12 (TNFSF12), transcript variant 2, mRNA	TNFSF12	ILMN_1680003	8742	−0.75	a
Serum amyloid A1 (SAA1), transcript variant 1, mRNA	SAA1	ILMN_1808732	6288	−1.29	a

a: *p* for interaction between intervention and group <0.05; b: *p* for intervention <0.05. Genes showed consistent expression change in obese subjects (>25 % up or >25 % down in at least 6 subjects)

### Data integration

We performed network analysis based on biological knowledge using MetaCore software to integrate the data, from plasma as well as adipose tissue.

**Fig. 2:**
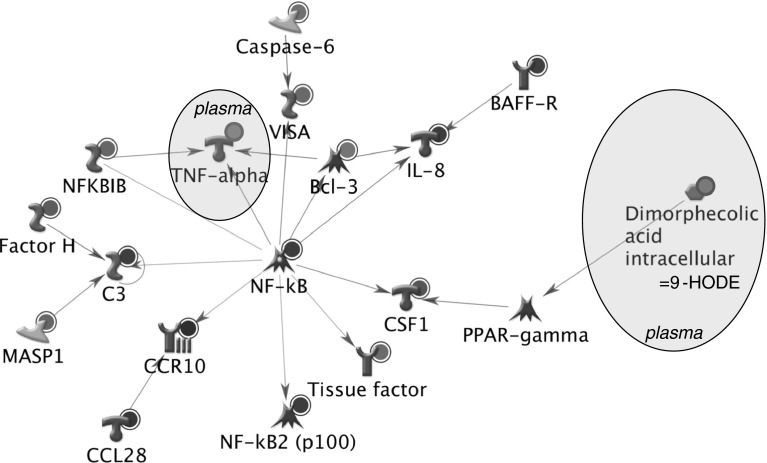
Network showing biological links between genes involved in inflammation and plasma markers that respond to high vegetable intake in lean subjects. *Red circle* indicates up-regulation in response to high vegetable intake, *blue circle* indicates down-regulation in response to high vegetable intake. *BAFF*-*R* tumor necrosis factor receptor superfamily, member 13C; *Bcl*-*3* B cell CLL/lymphoma 3; *C3* complement component 3; *CCL28* chemokine (C–C motif) ligand 28; *CCR10* chemokine (C–C motif) receptor 10; *CSF1* colony stimulating factor 1 (macrophage); *Factor H* complement factor H; *IL*-*8*: interleukin 8; *MASP1*: mannan-binding lectin serine peptidase 1 (C4/C2 activating component of Ra-reactive factor); *NF*-*kB*: nuclear factor-kappa-B; *NFKBIB* I-kappa-B-beta; *PPAR*-*γ* peroxisome proliferator-activated receptor gamma; *TNF*-*α* tumor necrosis factor alpha; *VISA* mitochondrial antiviral signaling protein

In lean subjects, high vegetable intake resulted in differential expression of 40 genes annotated to immune-related processes. The biological network in Fig. 2 shows direct interactions between 15 of these genes and plasma TNF-alpha, with transcription factor NFkB as a central regulator. Furthermore, network analysis showed that 9-HODE (measured in plasma) may be linked to this network through PPARγ. Potential anti-inflammatory effects of high vegetable intake in lean subjects are supported by down-regulation of NF-kB and target genes like tissue factor and IL-8 in adipose tissue as well as down-regulation of TNF-alpha levels in plasma.

**Fig. 3:**
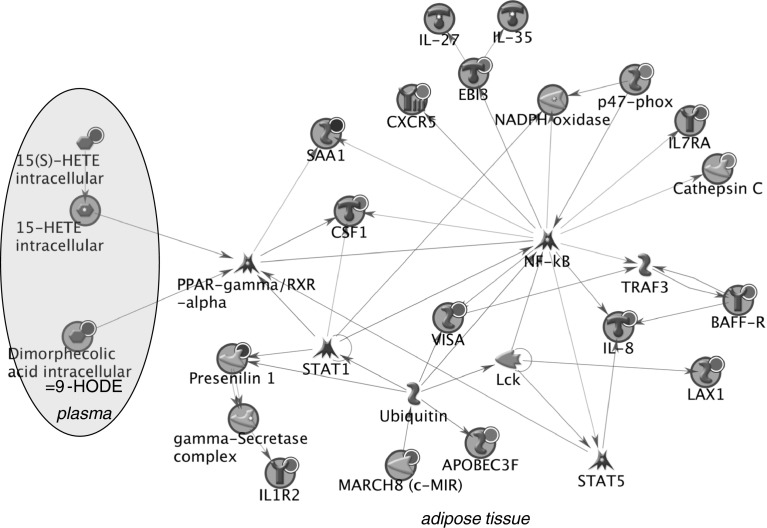
Network showing biological links between genes involved in inflammation and plasma markers that respond to high vegetable intake in obese subjects. *Red circle* indicates up-regulation in response to high vegetable intake, *blue circle* indicates down-regulation in response to high vegetable intake. *APOBEC3F*: apolipoprotein B mRNA editing enzyme, catalytic polypeptide-like 3F; *BAFF*-*R* tumor necrosis factor receptor superfamily, member 13C; *CSF1* colony stimulating factor 1 (macrophage); *EBIB* Epstein–Barr virus-induced 3, IL-27 subunit beta; *CXCR5* chemokine (C-X-C motif) receptor 5; *15(S)*-*HETE* 15-HETE; *IL1R2*: interleukin-1 receptor type 2; *IL*-*8* interleukin-8; *IL*-*27* interleukin-27; *IL*-*35* interleukin-35; *LAX1* lymphocyte transmembrane adaptor 1; *Lck* tyrosine-protein kinase Lck; *MARCH8 (c*-*MIR)* membrane-associated ring finger (C3HC4) 8; *NF*-*kB* nuclear factor-kappa-B; *p47*-*phox* neutrophil cytosolic factor 1; *PPAR*-*γ* peroxisome proliferator-activated receptor gamma; *RXR*-*α* retinoid X receptor, alpha; *SAA1* serum amyloid A1; *STAT1* signal transducer and activator of transcription 1; *STAT5* signal transducer and activator of transcription 5; *TRAF3* TNF receptor-associated factor 3; *VISA* mitochondrial antiviral signaling protein

**Fig. 4:**
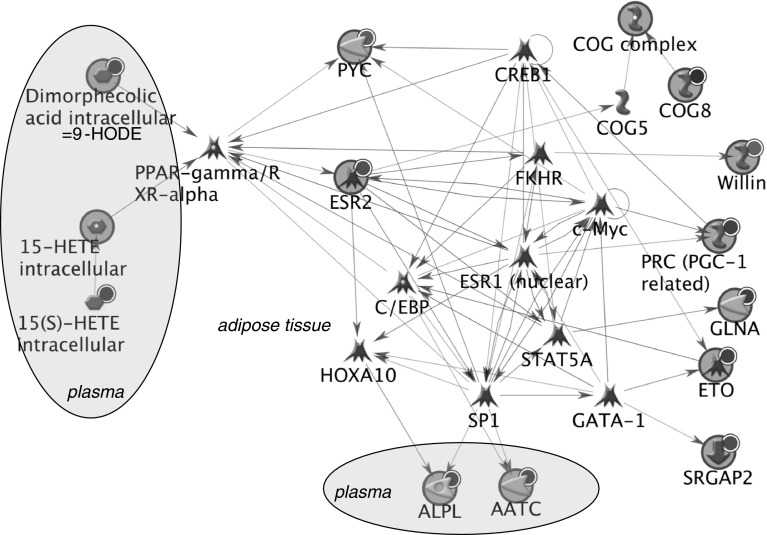
Network showing biological links between genes involved in energy metabolism and plasma markers that respond to high vegetable intake in obese subjects. *Red circle* indicates up-regulation in response to high vegetable intake, *blue circle* indicates down-regulation in response to high vegetable intake. *AATC* glutamic-oxaloacetic transaminase 1, soluble (aspartate aminotransferase 1); *ALPL* alkaline phosphatase, liver/bone/kidney; *C/EBP* CCAAT/enhancer binding protein (C/EBP); *COG complex* component of oligomeric golgi complex; *COG5* component of oligomeric golgi complex 5; *COG8* component of oligomeric golgi complex 8; *CREB1* cAMP responsive element binding protein 1; *c*-*Myc* v-myc myelocytomatosis viral oncogene homolog (avian); *ESR1 (nuclear)* estrogen receptor 1; *ESR2* estrogen receptor 2 (ER beta); *ETO* runt-related transcription factor 1; translocated to, 1 (cyclin D-related); *FKHR* forkhead box O1; *GATA*-*1* GATA binding protein 1 (globin transcription factor 1); *GLNA* glutamate-ammonia ligase; *15(S)*-*HETE* 15S-hydroxyeicosatetraenoic acid; *15*-*HETE* 15-hydroxyeicosatetraenoic acid; *HOXA10* homeobox A10; *PPAR*-*γ* peroxisome proliferator-activated receptor gamma; *RXR*-*α* retinoid X receptor, alpha; *PRC (PGC*-*1 related)* peroxisome proliferator-activated receptor gamma, coactivator-related 1; *PYC* pyruvate carboxylase; *SP1* Sp1 transcription factor; *SRGAP2* SLIT-ROBO Rho GTPase activating protein 2; *STAT5A* signal transducer and activator of transcription 5A; *Willin* FERM domain containing 6

To illustrate possible biological links between observed effects of high vegetable intake on obese subjects, biological networks on the basis of inflammation, energy metabolism and adhesion genes from Table 4 together with the significantly changed plasma metabolites and classical markers were created. The network in Fig. 3 shows that the inflammatory gene expression changes due to vegetable intervention in adipose tissue may be regulated by NFkB and PPARγ and that 9-HODE and 15-HETE (measured in plasma) may be linked to this network through PPARγ. All but two inflammatory genes listed in Supplementary File 2 are connected in the network. Furthermore, the network in Fig. 4 illustrates a biological link between 9-HODE and 15-HETE and specific energy metabolism genes, also through PPARγ. In addition to PPARγ, many other transcription factors may be involved in the regulation of energy metabolism in obese subjects, for example, CREB1, STAT5A, ESR2, ESR1 and SP1. Through these regulators, the plasma markers ASAT (AATC) and ALP (ALPL) were also linked to this network.

Biological network analysis did not reveal a link between the expression changes of adhesion genes and the plasma biomarker changes (see Supplementary File 3).

## Discussion

By integration of sensitive omics technologies with classic and modern metabolome biomarkers, we were able to show health effects of increased vegetable intake in both lean and obese subjects and elucidate some of the underlying mechanisms. The responsive markers included classical markers ASAT and ALP, plasma amino acids and oxylipins quantified by metabolic profiling as well as adipose tissue gene expression. The latter results suggest that both vegetable intake and weight reduction is differently regulated in lean and obese subjects. Data integration through network analysis illustrated a central role for NFkB in (adipose tissue) modulation of inflammation by increased vegetable intake, in lean as well as obese subjects. Plasma inflammatory cytokines did not change in response to increased vegetable intake, with the exception of TNF-α that showed lower concentrations in lean subjects. In obese subjects, high vegetable intake also resulted in adipose tissue gene expression changes related to energy metabolism and cell adhesion.

Subjects consumed both fresh and canned vegetables; the types of vegetables offered were the most popular types: (fresh) tomatoes, cauliflower, beet, onions; (canned) peas, broad beans, French beans, carrots, corn. Body weight remained stable through both high and low vegetable intakes. The finding that the highest treatment dose of 200 g of vegetables daily affects physiological processes in a direction that is associated with health benefits supports the advice on the Dutch Health Council to consume 200 g of vegetables daily (Health Council of the Netherlands 2006). Higher dosages of vegetables could scientifically be even more interesting; however, this will be considerably challenging for public health implementation and probably not yet realistic. In public surveys, it was found that on average about 2 % of the Dutch population consume 150 g of vegetables daily, and therefore, even 200 g daily may already provide a challenging target.

In the present study investigating health effects of vegetables, an energy restriction intervention was included to determine whether similar processes are present and similar effects on health were found. Although the subjects did not lose a lot of weight, still beneficial health effects were seen at the level of classical markers including weight loss, decreases in total cholesterol, LDL cholesterol, ratio of cholesterol/HDL and HbA1c. It was already known that about 5 % weight loss is beneficial for health (Wing et al. 1992; Tuomilehto et al. 2001; Calder et al. 2011). We report in the present study that 2 % body weight loss significantly affects established biomarkers of (cardiovascular) health.

After 8 weeks of prescribed vegetable consumption, subjects were instructed to eat in total less energy. The energy-restricted diet intervention was deliberately designed at the end of the study for all subjects, because we expected clear physiological impact due to energy restriction. This could not be implemented during the low/high vegetable intake intervention because it would have influenced the differences in vegetable intake. As a period *ad random* in the crossover trial, it would have affected the vegetable interventions too much, and therefore, only the vegetable interventions were designed as a crossover with an energy-restricted period afterward for all subjects.

In contrast to the energy restriction intervention, plasma markers did not show clear health benefits due to increased vegetable consumption. Two liver enzymes, ALP and ASAT, showed decreased concentrations after 4 weeks of 200 g of vegetable consumption. In lean subjects, the TNF-α level decreased with high vegetable consumption. The sensitive metabolic profiling analyses showed additional health effects. High vegetable intake resulted in significantly increased plasma levels of four amino acids (derivatives): asparagine, leucine, methionine sulfoxide and threonine. The amino acids may directly originate from the consumed vegetables and thus leading to increased plasma levels. So far, only O’Sullivan reported on dietary intake patterns among which vegetable consumption and their reflection in plasma and urinary metabolic profiles (O’Sullivan et al. 2011). Unfortunately, nothing was reported on vegetable intake and their plasma reflection on amino acids, so therefore, this finding could not be confirmed. Methionine sulfoxide is a well-known biomarker for oxidative stress, which occurs by oxidation of methionine residues to methionine sulfoxide (Mashima et al. 2003). It can be produced in vivo, but it is also known that plants produce this metabolite in response to stress (Emes 2009), since the relative response of this plasma metabolite to an a maximal exercise challenge is significantly lower in the subjects on high vegetables compared to low vegetables in this study, which was interpreted that high vegetables protected subjects from the acute in vivo damage by oxidative stress (results not shown). Furthermore, it is known that processing of foods can lead to modifications of proteins by oxidation, such as methionine sulfoxide (Rutherfurd and Moughan 2008). Taken together, we interpreted the significant increase in plasma methionine sulfoxide concentrations likely to be due to the higher intake of processed vegetables. Levels of lipid-derived metabolites 9-HODE, 15-HETE and prostaglandin D3 decreased in response to the 200-g vegetable intake compared to 50-g vegetable intake. 9-HODE and 15-HETE are both reduced products synthesized from, respectively, linoleic acid and arachidonic acid in the 12- and 15-lipoxygenase (LOX) pathways. These pathways are associated with LDL oxidation and subsequently with atherosclerotic lesions (Imaizumi et al. 2010). 9(S)-HODE and 13(S)-HODE are known as lipid peroxidation markers that can be used for the estimation of oxidative stress in human (Spiteller and Spiteller 1997). The oxylipin 13(S)-HODE showed a trend for treatment effect (*p* value 0.0543) with similar direction as 9-HODE and 15-HETE. Therefore, decreased concentrations of products from the 12- and 15-LOX pathways suggest decreased oxidative stress in subjects in response to high vegetable intake. This illustrates that subtle, beneficial processes take place and can be shown with this technology when classical markers do not show any effect.

In addition to the plasma metabolic profiling, we studied effects of the nutritional interventions in adipose tissue by whole-genome expression profiling. Previously, we and others have shown that nutritional intervention can result in adipose tissue gene expression changes (van Erk et al. 2008; Radonjic et al. 2009), including effects on inflammatory processes. Furthermore, it is expected that energy restriction and body weight loss result in changes in adipose tissue, specifically in obese subjects (Márquez-Quiñones et al. 2010; Bouchard et al. 2010). In obese subjects, weight loss due to the energy restriction was associated with changes in processes related to energy metabolism: oxidative phosphorylation, mitochondrial function, generation of precursor metabolites and energy. Also, process of adhesion and T cell activation (inflammation) were changed in concordance with weight loss. These processes are also reported in studies investigating adipose tissue response to very-low-calorie diets in obese subjects (Clément et al. 2004; Franck et al. 2011).

High vegetable intake resulted in subtle gene expression changes in adipose tissue. We selected differentially expressed genes based on statistics combined with a fold change threshold (>25 % up or >25 % down in at least 6 subjects). This approach takes into account inter-individual variation in response while still identifying group effects of a certain effect size. By submitting the selected sets of differentially expressed genes to enrichment analysis, we could identify the biological processes affected by increased vegetable intake. In lean subjects, increased vegetable consumption affected adipose tissue inflammation, as measured by gene expression. Through network analysis based on biological knowledge, we could visualize direct links between 15 adipose tissue genes with a role in inflammation. These genes were directly linked to TNF-alpha, for which plasma levels were decreased by increased vegetable intake in lean subjects. Within the group of obese subjects, increased vegetable intake resulted in a smaller set of differentially expressed genes that was not enriched for biological processes. This may be due to a larger variation in response to the obese group of subjects. We have seen such a larger response variation (factor two different) in a previous study as well (van Erk et al. 2008).

Weight loss is a well-accepted health benefit for obese subjects (Wing et al. 1992; Tuomilehto et al. 2001; Calder et al. 2011). By correlating adipose tissue gene expression changes to body weight change after caloric restriction, we identified pathways or processes that are associated with this health benefit. These processes were only identified in the obese subjects. This makes sense as these subjects, being overweight, would benefit most from weight loss. In obese subjects, high vegetable intake resulted in differential expression of small sets of genes in all three processes: adhesion, energy metabolism and inflammation. In order to visualize biological links between these adipose tissue genes, plasma proteins and plasma metabolites, biological network analysis was performed The plasma metabolites related to amino acids were included in the network but did not appear to be involved in the inflammation processes. The network of inflammation genes revealed a central role of NFkB as regulator of inflammatory gene expression changes in adipose tissue due to high vegetable intake. Therefore, it could be hypothesized that in both lean and obese subjects, increased vegetable intake affects inflammatory processes in adipose tissue through NFkB. Also, the networks show a link of these adipose tissue inflammatory responses with decreases in plasma oxylipins 9-HODE and 15-HETE, through PPARγ/RXRα. These oxylipins are known to play a role in oxidative stress.

The network of energy metabolism genes also shows a connection to plasma oxylipins through PPARγ/RXRα. This network includes a range of transcription factors that could be involved rather than one central regulator for inflammation. Interestingly, this network includes ASAT (AATC) and ALP (ALPL), indicating a potential biological link between these markers and adipose tissue gene expression changes related to energy metabolism.

The network of adhesion genes did only reveal a link to plasma ALP and not to other plasma markers or central regulators.

Network analysis has the advantage of combined analysis of markers from different levels (gene, protein, metabolite) and of focus on biological connections between markers that show a significant response. The networks provide hypotheses of possible effects and mechanisms of health-promoting effects of high vegetable intake that can be investigated in more detail.

## Conclusion

By inclusion of sensitive omics technologies and comparing the changes induced by high vegetable intake with changes induced by energy restriction and associated with weight loss, it has been shown that part of vegetables’ health benefits are mediated by changes in energy metabolism, inflammatory processes and oxidative stress.

## Electronic supplementary material

Below is the link to the electronic supplementary material.

Supplementary material 1 (XLSX 13 kb)

Supplementary material 2 (DOCX 19 kb)

Supplementary material 3 (PPTX 322 kb)
