# Supplementary material for: Nutrigenomics approach elucidates health-promoting effects of high vegetable intake in lean and obese men
Source: Genes Nutr. 2013 Apr 18;8(5):507–21. doi: 10.1007/s12263-013-0343-9 (PMC3755133; doi:10.1007/s12263-013-0343-9)
Supplement: Supplementary file 2 — Supplementary material 2 (DOCX 19 kb) [file 12263_2013_343_MOESM2_ESM.docx]

**Supplementary file 2.**

Table listing differential gene expression changes in response to high vegetable intake compared to low vegetable in take in lean subjects

|  |  |  |  | LEAN |  |
| --- | --- | --- | --- | --- | --- |
| **Probe_Id** | **Definition** | **Symbol** | **Entrez_Gene_**  **ID** | **mean 2logratio** |  |
| ILMN_2338452 | serpin peptidase inhibitor, clade A (alpha-1 antiproteinase, antitrypsin), member 1 (SERPINA1), transcript variant 2, mRNA. | SERPINA1 | 5265 | -1.31 | acute inflammatory response |
| ILMN_1666966 | insulin (INS), mRNA. | INS | 3630 | -0.84 | acute inflammatory response, regulation of lipid metabolic process |
| ILMN_1680714 | ligase IV, DNA, ATP-dependent (LIG4), transcript variant 2, mRNA. | LIG4 | 3981 | -0.73 | immune response |
| ILMN_2121568 | guanylate binding protein family, member 6 (GBP6), mRNA. | GBP6 | 163351 | -0.72 | immune response |
| ILMN_1666493 | chemokine (C-C motif) receptor 10 (CCR10), mRNA. | CCR10 | 2826 | -0.71 | chemokine signaling pathway |
| ILMN_1697309 | neutrophil cytosolic factor 1 (NCF1), mRNA. | NCF1 | 653361 | -0.66 | immune response, chemokine signaling pathway |
| ILMN_2228845 | chemokine (C-C motif) ligand 28 (CCL28), mRNA. | CCL28 | 56477 | -0.65 | immune response, chemokine signaling pathway |
| ILMN_1674574 | vanin 1 (VNN1), mRNA. | VNN1 | 8876 | -0.63 | immune response, acute inflammatory response, cell adhesion |
| ILMN_2184373 | interleukin 8 (IL8), mRNA. | IL8 | 3576 | -0.61 | immune response, chemokine signaling pathway, inflammatory response |
| ILMN_1732919 | leukocyte immunoglobulin-like receptor, subfamily B (with TM and ITIM domains), member 5 (LILRB5), transcript variant 2, mRNA. | LILRB5 | 10990 | -0.61 | immune response |
| ILMN_2390859 | nuclear factor of kappa light polypeptide gene enhancer in B-cells 2 (p49/p100) (NFKB2), transcript variant 2, mRNA. | NFKB2 | 4791 | -0.61 | immune response |
| ILMN_1731742 | tumor necrosis factor receptor superfamily, member 13C (TNFRSF13C), mRNA. | TNFRSF13C | 115650 | -0.60 | immune response |
| ILMN_1655935 | adenylate cyclase 7 (ADCY7), mRNA. | ADCY7 | 113 | -0.57 | chemokine signaling pathway |
| ILMN_2284794 | proteasome (prosome, macropain) subunit, beta type, 8 (large multifunctional peptidase 7) (PSMB8), transcript variant 1, mRNA. | PSMB8 | 5696 | -0.56 | immune response |
| ILMN_2415786 | CD96 molecule (CD96), transcript variant 2, mRNA. | CD96 | 10225 | -0.53 | immune response, cell adhesion |
| ILMN_1699160 | IL2-inducible T-cell kinase (ITK), mRNA. | ITK | 3702 | -0.51 | chemokine signaling pathway |
| ILMN_2167416 | major histocompatibility complex, class I-related (MR1), mRNA. | MR1 | 3140 | -0.50 | immune response |
| ILMN_1680996 | PREDICTED: arachidonate 5-lipoxygenase (ALOX5), mRNA. | ALOX5 | 240 | -0.50 | inflammatory response |
| ILMN_1808846 | TBK1 binding protein 1 (TBKBP1), mRNA. | TBKBP1 | 9755 | -0.47 | immune response |
| ILMN_1808405 | PREDICTED: major histocompatibility complex, class II, DQ alpha 1, transcript variant 10 (HLA-DQA1), mRNA. | HLA-DQA1 | 3117 | -0.45 | immune response |
| ILMN_1765668 | interleukin 20 receptor beta (IL20RB), mRNA. | IL20RB | 53833 | -0.45 | immune response, inflammatory response |
| ILMN_1669674 | canopy 3 homolog (zebrafish) (CNPY3), mRNA. | CNPY3 | 10695 | -0.40 | immune response |
| ILMN_1722622 | CD163 molecule (CD163), transcript variant 2, mRNA. | CD163 | 9332 | -0.38 | acute inflammatory response |
| ILMN_1797009 | coagulation factor III (thromboplastin, tissue factor) (F3), mRNA. | F3 | 2152 | -0.30 | acute inflammatory response |
| ILMN_2337931 | chemokine (C-X-C motif) receptor 5 (CXCR5), transcript variant 2, mRNA. | CXCR5 | 643 | 0.29 | chemokine signaling pathway |
| ILMN_1807283 | N-deacetylase/N-sulfotransferase (heparan glucosaminyl) 1 (NDST1), mRNA. | NDST1 | 3340 | 0.42 | inflammatory response |
| ILMN_1736426 | phospholipase C, beta 4 (PLCB4), transcript variant 2, mRNA. | PLCB4 | 5332 | 0.43 | chemokine signaling pathway |
| ILMN_1671237 | guanine nucleotide binding protein (G protein), gamma transducing activity polypeptide 2 (GNGT2), mRNA. | GNGT2 | 2793 | 0.47 | chemokine signaling pathway |
| ILMN_1710514 | B-cell CLL/lymphoma 3 (BCL3), mRNA. | BCL3 | 602 | 0.48 | immune response |
| ILMN_1694877 | caspase 6, apoptosis-related cysteine peptidase (CASP6), transcript variant alpha, mRNA. | CASP6 | 839 | 0.51 | acute inflammatory response |
| ILMN_2131493 | virus-induced signaling adapter (VISA), mRNA. | VISA | 57506 | 0.59 | immune response, mitochondrion |
| ILMN_2089875 | tumor necrosis factor (ligand) superfamily, member 4 (tax-transcriptionally activated glycoprotein 1, 34kDa) (TNFSF4), mRNA. | TNFSF4 | 7292 | 0.61 | immune response, inflammatory response |
| ILMN_1690920 | SP100 nuclear antigen (SP100), transcript variant 2, mRNA. | SP100 | 6672 | 0.62 | immune response |
| ILMN_1674152 | nuclear factor of kappa light polypeptide gene enhancer in B-cells inhibitor, beta (NFKBIB), transcript variant 1, mRNA. | NFKBIB | 4793 | 0.64 | chemokine signaling pathway |
| ILMN_1801996 | mannan-binding lectin serine peptidase 1 (C4/C2 activating component of Ra-reactive factor) (MASP1), transcript variant 3, mRNA. | MASP1 | 5648 | 0.64 | immune response, acute inflammatory response |
| ILMN_1778536 | B and T lymphocyte associated (BTLA), transcript variant 1, mRNA. | BTLA | 151888 | 0.71 | immune response |
| ILMN_1677920 | lactotransferrin (LTF), mRNA. | LTF | 4057 | 0.73 | immune response |
| ILMN_1698144 | complement factor H (CFH), transcript variant 1, mRNA. | CFH | 3075 | 0.74 | immune response, acute inflammatory response |
| ILMN_1805930 | colony stimulating factor 1 (macrophage) (CSF1), transcript variant 4, mRNA. | CSF1 | 1435 | 0.83 | immune? |
| ILMN_1662523 | complement component 3 (C3), mRNA. | C3 | 718 | 1.07 | immune response, acute inflammatory response |
