# Supplementary material for: Nutrigenomics approach elucidates health-promoting effects of high vegetable intake in lean and obese men
Source: Genes Nutr. 2013 Apr 18;8(5):507–21. doi: 10.1007/s12263-013-0343-9 (PMC3755133; doi:10.1007/s12263-013-0343-9)
Supplement: Supplementary file 3 — Supplementary material 3 (PPTX 322 kb) [file 12263_2013_343_MOESM3_ESM.pptx]

## Slide 1
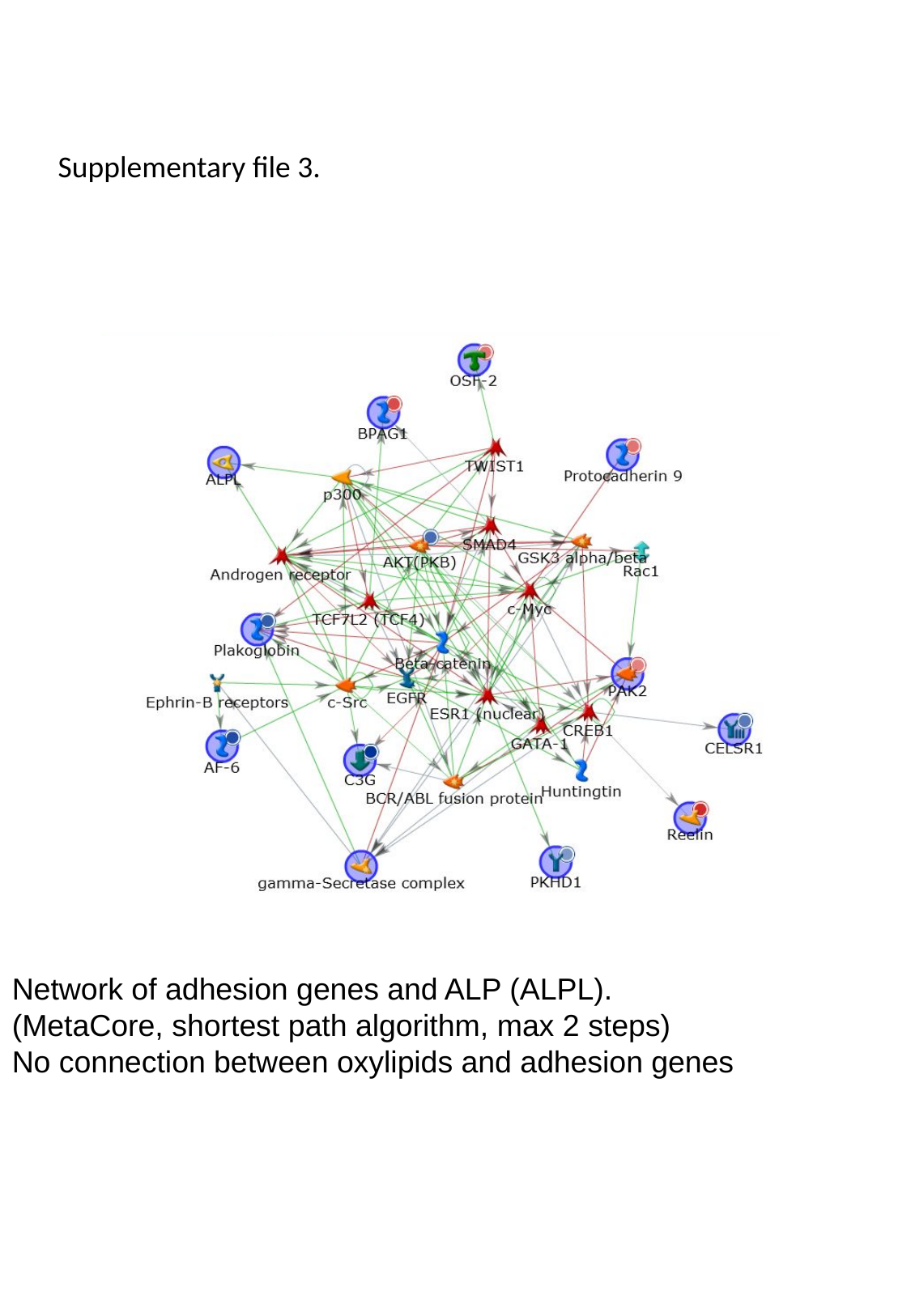

Supplementary file 3.
Network of adhesion genes and ALP (ALPL).
(MetaCore, shortest path algorithm, max 2 steps)
No connection between oxylipids and adhesion genes
